# Optimizing intraluminal monofilament model of ischemic stroke in middle-aged Sprague–Dawley rats

**DOI:** 10.1186/s12868-022-00764-2

**Published:** 2022-12-09

**Authors:** I. J. Biose, W. H. Chastain, H. Wang, B. Ouvrier, G. J. Bix

**Affiliations:** 1 Department of Neurosurgery, Clinical Neuroscience Research Center, Tulane University School of Medicine, New Orleans, LA 70112 USA; 2 School of Medicine, Tulane University, New Orleans, LA 70112 USA; 3 Tulane Brain Institute, Tulane University, New Orleans, LA 70112 USA; 4 Department of Neurology, Tulane University School of Medicine, New Orleans, LA 70112 USA; 5 Department of Microbiology and Immunology, Tulane University School of Medicine, New Orleans, LA 70112 USA; 6 Tulane University School of Public Health and Tropical Medicine, New Orleans, LA 70122 USA

**Keywords:** Monofilament, Doccol filament, MCAO, Sprague–Dawley rats, Middle age

## Abstract

Intraluminal monofilament model of middle cerebral artery occlusion (MCAO) is widely adopted for ischemic stroke; and Sprague–Dawley (SD) rats are commonly used rodents for preclinical research. Due to the paucity of information on the appropriate monofilament size for inducing MCAO in SD rats and the importance of including middle-aged models in ischemic stroke studies, we aimed to: (i). determine an appropriate Doccol^®^ monofilament size for middle-aged male SD rats which weighed > 500 g following 24-h transient MCAO survival as well as (ii). demonstrate the optimal Doccol^®^ filament size for middle-aged males (≤ 500 g) and females (273–300 g) while using young adult male SD rats (372–472 g) as control for severity of infarct volume following 7-days post-MCAO. All rats were subjected to 90-min transient MCAO. We show that 0.43 mm Doccol^®^ monofilament size is more appropriate to induce large infarct lesion and optimal functional deficit when compared to 0.45 mm and 0.47 mm at 24 h post-MCAO. Our data on infarct volumes at 7 days post-MCAO as well as the observed weight loss and functional deficits at post-MCAO days 1, 3 and 7 demonstrate that 0.41 mm, 0.37 mm and 0.39 mm are optimal Doccol^®^ filament sizes for middle-aged male (477.3 ± 39.61 g) and female (302.6 ± 26.28 g) as well as young-adult male (362.2 ± 28.38 g) SD rats, respectively.

## Introduction

Stroke is a common cerebrovascular disease and a significant cause of debilitating disability and death [1]. Indeed, over 12 million new stroke cases are reported each year [1, 2], making stroke of significant public health concern and aptly justifies ongoing intensive preclinical research to increase knowledge and investigate potential therapies. Although stroke is a disease of the elderly, a significant number of stroke patients are middle-aged adults [1, 3, 4]. This may be due to a higher prevalence of stroke risk factors such as hypertension, diabetes, and dyslipidemia in this population [5–7].

Ischemic stroke, which accounts for 87% of all stroke cases in the United States [1], results from blockage of blood supply to brain tissues, causing brain cell death and loss of bodily function(s) to ensue. With the availability and access to advanced treatments such as thrombolytics and mechanical thrombectomy being associated with reduced ischemic stroke mortality [8], the incidence of ischemic stroke at midlife (45–65 years of age) is unaffected or increasing in some subpopulations [6, 7, 9]. This indicates that the number of individuals who will live with the debilitating loss of bodily function is on the rise. Also, individuals living with stroke at midlife grapple with peculiar challenges which lowers their quality of life [7]. These challenges often range from adaptations for returning to work, loss of employment, dependence for selfcare, increased cost of healthcare for continuing physical and psychological therapies [10–12], to substantial risks for recurrent stroke which is associated with worsened loss of bodily function and increased mortality [13, 14]. As the number of midlife stroke survivors increases, the potential loss of workforce as well as the overall socio-economic impacts of stroke at middle-age will worsen. Hence, it is important to continue intensified efforts towards translational research for midlife stroke.

To advance knowledge on mechanisms as well as investigate potential therapies, ischemic stroke is modeled in rodents. Essentially, the middle cerebral artery (MCA) is occluded using various strategies reviewed elsewhere [15]. Intraluminal monofilament model is a commonly used model to study ischemic stroke in rats. While some monofilaments are custom made, the majority of researchers use the commercially available Doccol^®^ monofilament for its silicon tip which adheres to the vascular endothelium and ensure the occlusion of the MCA in rodents [16, 17]. However, Doccol^®^ only provides suggestions for filament diameter on the basis of body weight range. In our experience, the recommended monofilament size does not always correspond with infarct size observation. For example, 4039356PK10 monofilament is recommended for rodents weighing 320 ± 20 g, but we have successfully used this for young adult male Sprague–Dawley (SD) rats weighing 400 ± 50 g. Also, we [18] have successfully used 50-3033PKRe monofilament to induce MCAO in 300–365 g spontaneously hypertensive stroke prone rats, even though Doccol^®^ recommends their use for rodents weighing 200–250 g.

Further, SD rats are commonly used preclinical models [19] by reason of cheaper cost, commercial availability, and translational relevance. Given the translational importance of considering middle age in the investigation of potential therapies for ischemic stroke as well as the paucity of data on intraluminal monofilament model of MCAO used in middle aged SD rats, we aimed to determine an appropriate filament size for overweight (> 500 g) middle aged male SD rats to induce ischemic stroke. Nevertheless, neurological deficit, with or without measurable infarct volume, is the ultimate target of ischemic stroke outcome and 24 h is too early to evaluate functional outcome. Hence, we report the appropriate monofilament size for common weight range of middle-aged male and female SD rats by comparing infarct volume, neurological and body weight changes with those of young adult male SD rats following a 7-day post-reperfusion survival experiment.

## Materials and methods

### Animals, study groups and approval

A total of sixty-two SD rats (12–13 months old middle-aged males [408–641 g] and females [279.5–336 g] as well as young adult males [9–12 weeks old; 330–404.5 g]) were allowed at least two weeks of acclimatization following delivery from Envigo^®^, USA to Tulane University’s animal housing facility. For study 1, 12–13 months old SD rats which weighed > 500 g (i.e. 519–641 g) were randomly assigned to three monofilament size groups: 0.43 mm group, n = 12; 0.45 mm group, n = 12; and 0.47 mm group, n = 11. For study 2, all other rats were allocated to one of three groups, according to their sex and age: i. middle aged males (408.3–531.4 g), n = 9, ii. Middle aged females (280–336 g), n = 9, and iii. young adult males (330–405 g), n = 9.

All rats were maintained in identical housing conditions: no more than two per cage, standard 12 h light–dark cycle, food and water ad libitum. All animal procedures were approved by Tulane University Institutional Animal Care and Use Committee. Experimental procedures, data analysis, documentation and reporting were conducted in accordance to ARRIVE guidelines 2.0 (https://arriveguidelines.org/arrive-guidelines). All methods were performed in accordance with the relevant Institutional approval and ARRIVE guidelines 2.0.

### Middle cerebral artery occlusion (MCAO)

The induction of 90 min transient occlusion of the proximal MCA, using a minor modification of the intraluminal monofilament model of Koizumi [20], was performed by aseptic surgical technique. Surgical plane of anesthesia was achieved with the delivery of 2–2.5% isoflurane carried in oxygen and nitrous oxide (O_2_, N_2_O: 0.3L/min, 0.7L/min) following an initial anesthetic induction with 5% isoflurane. Adequate anesthesia was confirmed by absence of hind-limb withdrawal reflex. To prevent corneal dryness, eye ointment (Artificial tears, Akorn Inc., USA) was applied to both eyes before and after surgical procedures. The fur over the surgical sites of the ventral neck and above the left zygomatic arch was removed by depilatory cream (Nair™, Church and Dwight Co Inc., USA) before transfer to the surgical table with a heating pad to maintain physiological body temperature (36.5–37.5 ℃, temperature controller, Harvard Apparatus, USA). Prior to incision, the skin was disinfected using alcohol and betadine, and ropivacaine block (1–2 mg/kg s.c., NDC17478-081-30, Akorn operating company LLC, USA) was administered at the sites of incision.

An incision in the ventral neck and careful separation of the salivary glands was made to expose the common carotid artery (CCA). The external (ECA) and internal (ICA) carotid arteries were isolated superior to the CCA. Using 4–0 silk suture, ligatures were placed at the proximal portion of the CCA (permanent knot) and another (loose knot) below CCA bifurcation but above the ventral cut to prevent blood loss. A 4–0 nylon monofilament with silicone coated tip (404756PK10, 404556PK10 or 404356PK10 for middle aged males in study 1; 404156PK10 for middle aged males, 403756PK10 for middle aged females and 403956PK10 for young adult males in study 2; Doccol Corporation, MA, USA) was inserted into the ventral cut on the CCA and advanced through the ICA, to occlude the origin of the MCA. Each rat was recovered from anesthesia in a pre-warmed cage for 90 min prior to reperfusion. Upon reperfusion of the MCA, the monofilament was removed to re-establish blood flow. Cerebral perfusion was measured prior to and following the induction of MCA occlusion (MCAO) as well as after reperfusion, using Laser Doppler Flowmetery (PeriFlux system 5000, Perimed AB, Sweden); by placing the single point laser probe just above the zygomatic arch between the left eye and ear. Following MCAO induction, all included animals had no less than 70% CBF reduction from baseline. Prior to reperfusion, exclusion criteria was set at ≤ 40% CBF reduction from baseline.

A splash block of the neck muscles (with 1–2 mg/kg of ropivacaine) was administered prior to the closure of the neck and head incisions using 9-mm wound clips (CellPoint Scientific Inc., USA). Normal saline (0.5 mL, ICU Medical Inc., USA) and 4 mg/kg of ropivacaine were subcutaneously administered immediately after reperfusion and daily for up to three days post-surgery for hydration and analgesia, respectively. Additionally, moistened food pellets were provided in home cages, for the study duration, to encourage feeding and hydration. Rats were observed twice daily and weighed once daily following surgery to monitor welfare, health and activity.

### Functional outcome assessments

For study 1, all middle-aged rats were subjected to 10-point neuroscore test 24 h following reperfusion. All rats in study 2 were subjected to functional tests on days -1 (baseline), 1, 3 and 7 post-surgery to evaluate changes in muscle strength, asymmetry of the sensorimotor cortex and striatal as well as motor coordination by use of grip-strength test, vibrissae-evoked forelimb placement and sticky dot tests, respectively. Prior to the commencement of each functional test, rats were allowed to acclimate to the test room for 1 h on each testing day.

#### 10-point neuroscore test

Neuroscore test is used to assess the severity of neurological impact of MCAO in rodents. The severity of assessed functions ranges from 10 points (which is scored for normal neurological function) to 0 point (which is scored for very poor neurological function) as enumerated in Table 1: Spontaneous movement (in cage), type of movement (out of cage), response to touch stimuli, and forelimb separation.

**Table 1:** 10-point neuroscore test

Parameter	Response	Points
Activity (3 min, in cage)	No movement	0
	Minimal movement/ movement following touch	1
	Touches 1–2 walls of home cage	2
	Touches 3–4 walls of home cage	3
Type of movement (out of the cage)	Spontaneous spins/rolling	0
	Spins when lifted by the tall	1
	Circles to one side while moving	2
	No circling/moves straight	3
Side stroking (maximum of 2 strokes)	No response	0
	Unilateral response	1
	Bilateral response	2
Forelimb extension	Unilateral forelimb forward extension	1
	Bilateral forelimb forward extension	2

To evaluate spontaneous movement in cage, the lid of the cage is removed, and individual animals are observed for 3 min. Rats which touch 3–4 walls of the cage are assigned 3 points while rats which only touch 1–2 cage walls and rats which moved only when touched by the tail or no movement observations are assigned 2, 1 or 0 points, respectively. Similarly, type of movement out of cage is assessed when the animal is allowed to move freely on a bench top for 60 s and lifted by the tail for 10 s. A straight moving rat is assigned 3 points while a rat which moves in circles to one side is assigned 2 points, rats which spins when lifted by the tail is assigned 1 point and any rat which was observed to roll or spin spontaneously is scored 0 point. Side stroking assessment is used to assess sensorimotor function by using the tip of a cotton bud to stroke the lateral sides of the rats, once. The movement of the rat towards or away from touch on both sides (bilateral response) is assigned 2 points. When a rat fails to move towards or away from the cotton bud following a side stroke on only one side (unilateral response) such rat was scored 1 point and when rats do not respond to stroking on either side, 0 point is assigned. Lastly, forelimb extension is used to assess motor function. Each rat was lifted by the tail for 10 s and if both forelimbs extend towards the side of the bench top, animals are assigned 2 points. However, if only one limb is extended forwards while the other limb is extended downwards, 1 point is assigned to the rat. Rats were allowed to rest for two minutes between each assessed parameter.

#### Grip strength test

Grip strength test is used to evaluate forelimb motor function and muscle strength following MCAO in rats. The careful lifting of each rat by the base of the tail allows both forepaws to be placed on the pull bar of the grip strength meter (Columbus Instruments, USA). Each rat was pulled by the tail towards the experimenter when the torso aligns horizontally with the pull bar and measured values are recorded per trial. Four trials were conducted on each test day per rat. Trials were performed by the same experimenter, with a relative consistency and force. Rats which failed to grasp the pull-bar with both paws were retested for that trial run. A zero score was assigned to any rat which failed to grab onto the pull bar with both forepaws, after eight attempts. Two minutes of rest was allowed between each trial, per rat.

#### Vibrissae-evoked forelimb placement test

Vibrissae-evoked forelimb placement test is used to evaluate asymmetry in the sensorimotor cortex and striatum of rodents. Each rat was held with one paw restrained against the chest wall while the paw on the impaired side hanged freely. Whiskers on the impaired side were brushed in a downward motion against the edge of a clean bench top. This process was performed for a total of ten trials per test day, with adequate rest time allowed, and the total number of ipsilateral paw contact with the edge of the bench is recorded.

#### Sticky dot test

Sticky dot test is used to assess forelimb motor co-ordination. Individual rats were carefully restrained to place white adhesive labels (3/4’’, Chromalabel^®^, USA) on the ventral side of both forepaws. Animals were then placed, individually, in a clean transparent plexi-glass chamber and allowed up to 300 s to remove the adhesive labels from both paws. The time taken to remove the adhesive label from the paw on the impaired side was recorded. Adhesive labels were removed for rats which did not successfully take off labels from their forepaw(s) after five minutes before returning to their home cage, and scored a maximum time of 300 s.

### Euthanasia, brain harvest, tissue sections and TTC stain

Twenty-four hours after reperfusion for study 1, and on day 7 post-MCAO for study 2, all rats were humanely sacrificed via decapitation following absence of pinch hindlimb reflex under isoflurane anesthesia. Harvested whole brain samples were rinsed in cold PBS and sectioned into 2 mm coronal slices using sectioning matrix (Kent Scientific Corporation, USA). Brain slices were incubated in warm 1% 2,3,5-triphenyltetrazolium chloride (TTC, Sigma, USA) for a total of 30 min (15 min per side) and sorted in a rostro-caudal manner for image acquisition using a 600-dpi resolution (HP scanner, G4050, USA). Image-J 1.80 (NIH, USA) was used to quantify infarct area. Infarct volume is calculated as the product of the summed infarct area and slice thickness. Swanson’s Edema Correction formulae was applied to adjust infarct volume of each slice [21].

### Data validation, blinding consideration and statistical analysis

Data analysis was conducted with Graphpad Prism 9.3 (GraphPad Software, USA) following validation by two independent researchers who were blind to the animal identifications. All data are presented as mean ± SD, as indicated in the figures. One-way ANOVA along with Tukey’s multiple comparisons test was used to analyze brain infarct volume. Repeated measures two-way ANOVA followed by Tukey’s multiple comparisons test was used to analyze body weight, grip strength, vibrissae score and sticky dot tests. Statistical significance for differences between groups was set at p < 0.05.

## Results

### 0.43 mm monofilament induces larger infarct volume in middle aged male SD rats > 500 g compared to 0.45 mm and 0.47 mm groups.

**Fig. 1:**
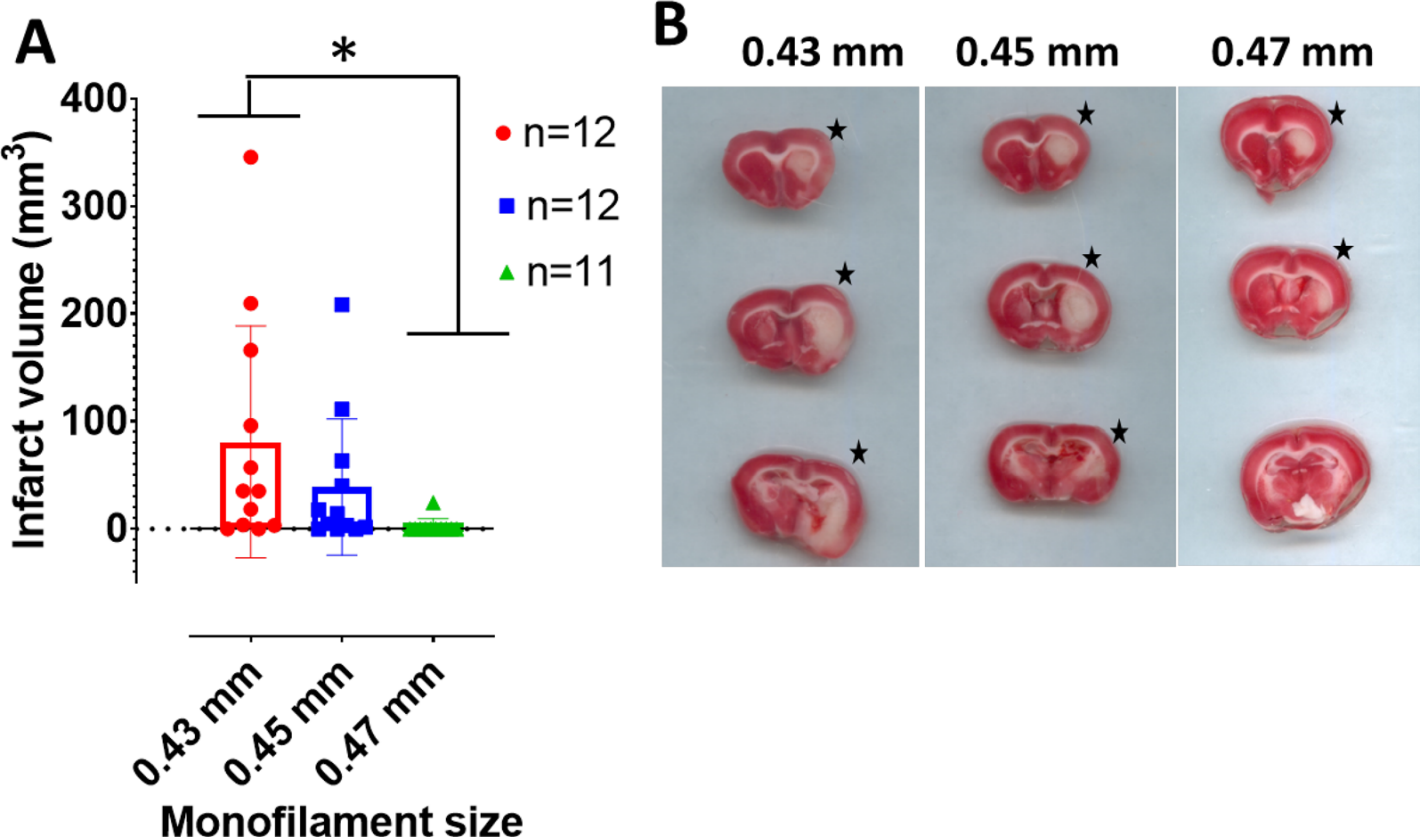
The use of 0.43 monofilament results in larger infarct volume in middle aged male SD rats > 500 g. **A** Infarct volume, 24 h following 90 min transient proximal MCAO (*p = 0.0391, one-way ANOVA with multiple comparisons) **B** Representative brain slices, per monofilament group, following 1% TTC staining 24 h post-MCAO. The black star denotes infarcted hemisphere per slice. Data presented as Mean ± SD

The effect of three monofilament sizes on infarct volume was assessed in three groups of middle-aged SD rats > 500 g. There was a statistically significant difference between treatment groups as shown by one-way ANOVA (F(2,32) = 3.312, p = 0.0493; Fig. 1A). Tukey’s multiple comparisons showed that monofilament size 0.43 mm significantly resulted in larger infarct volume, 24 h post MCAO, when compared to 0.47 mm monofilament (p = 0.0391; Fig. 1A). However, there is no statistically significant differences between 0.43 mm and 0.45 mm groups (p = 0.3492) as well as between 0.45 mm and 0.47 mm groups (p = 0.4669). Additionally, 0.43 mm and 0.45 mm monofilaments resulted in similar infarct sizes when compared to brains slices from the lone animal with infarct lesion in the 0.47 mm group (Fig. 1B).

### No difference in pre-reperfusion CBF; 0.43 mm reduces neurological function as well as body weight.

**Fig. 2:**
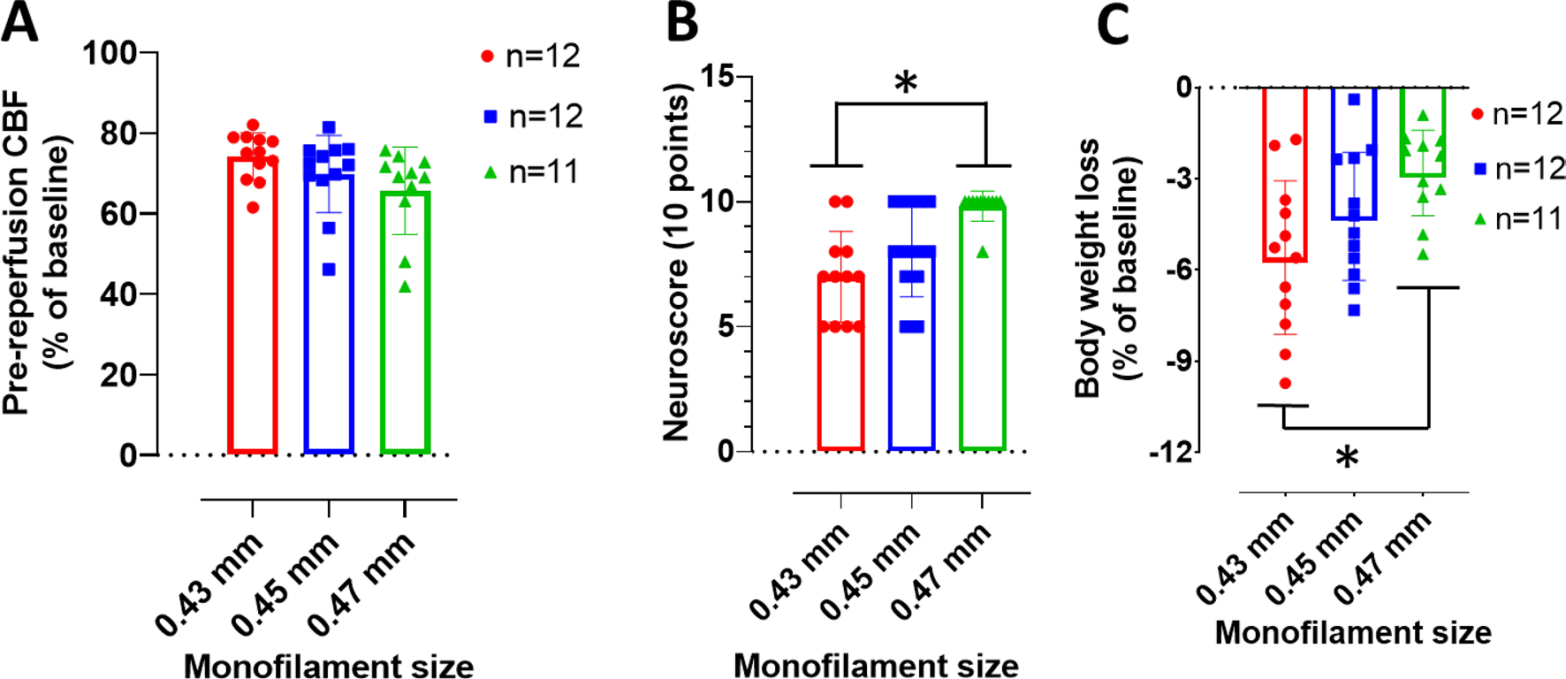
Pre-reperfusion cerebral blood flow (CBF) is comparable between groups but 0.43 mm severely impacts functional outcome and body weight. **A** Pre-reperfusion CBF following 90 min of MCAO and expressed as a percentage of baseline CBF. **B** 10-point neuroscore, 24 h following 90 min MCAO in middle aged male SD rats > 500 g. One-way ANOVA with multiple comparisons.** C** Change in body weight following 24 h of transient MCAO. Data presented as Mean ± SD, *p < 0.05

CBF values were assessed in all rats prior to the induction of MCAO (at baseline) as well as prior to reperfusion following 90 min of MCAO (pre-reperfusion). There is no statistically significant difference in pre-reperfusion CBF between the three monofilament groups as shown by one-way ANOVA (F(2,32) = 0.8477 and p = 0.0932; Fig. 2A). However, there is a monofilament size effect on neurological score following 24 h of transient MCAO in middle-aged SD rats > 500 g as indicated by One-way ANOVA (F(2,32) = 9.844 and p = 0.0005; Fig. 2B). The difference in neurological score between 0.43 mm an 0.47 mm groups is statistically significant as shown by Turkey’s comparison tests (p = 0.0391; Fig. 2B). Further, the difference between 0.43 mm and 0.45 mm groups is not statistically significant (p = 0.3492). Similarly, there is no statistically significant difference between 0.45 mm and 0.47 mm groups (p = 0.4669).

Change in body weight was assessed in the three monofilament groups following 24 h of transient MCAO. One-way ANOVA reveals a statistically significant difference between groups (F(2,32) = 5.128 and p = 0.0117; Fig. 2C). The difference between 0.43 mm and 0.47 mm groups is statistically significant (p = 0.0084; Fig. 2C). However, there is no statistically significant difference between 0.43 mm and 0.45 mm groups (p = 0.2605) as well as between 0.45 mm and 0.47 mm groups (p = 0.2448).

### Demonstrating optimal monofilament size in male and female SD rats

**Fig. 3:**
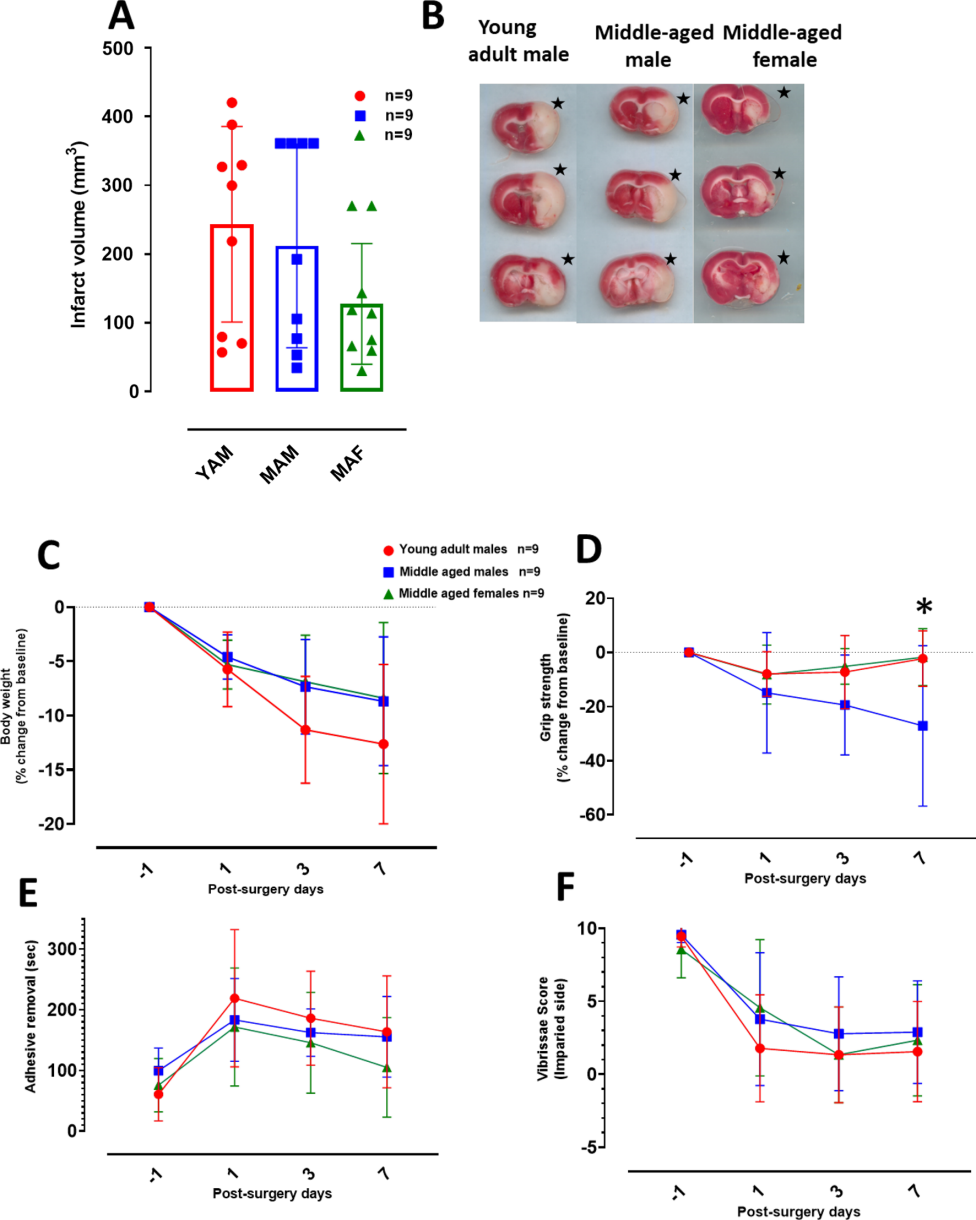
Infarct volume and functional outcome measures in middle aged and young adult SD rats. **A** Infarct volume, 7 days following 90 min transient proximal MCAO in young adult male (YAM), middle-aged male (MAM) and middle-aged female (MAF) SD rats. **B** Representative brain slices, per cohort, following 1% TTC staining 7 days after transient MCAO. Black star indicate infarcted hemisphere per slice.** C** Body weight change from baseline.** D** Grip strength change from baseline.** E** Adhesive removal time.** F** Vibrissae score on the impaired side. Data presented as Mean ± SD; *p < 0.05

The infarct volume was measured on day 7 following 90 min transient MCAO induction to demonstrate that 0.39 mm, 0.41 mm and 0.37 mm monofilaments are optimal for MCAO induction in young adult male, middle aged male and middle-aged female SD rats, respectively. The stated monofilament sizes induced unilateral infarct lesion with no statistically significant difference between groups (one-way ANOVA (F(2,24) = 1.939 and p = 0.1658; Fig. 3A). However, we note that while infarct size on brain slices is visually comparable between young adult males and middle-aged males, middle aged females have smaller infarct lesions as shown in Fig. 3B.

Body weight was measured at baseline, day 1, 3 and 7 post-MCAO to assess physiological welfare. There is an observed body weight loss on all measurement days post-MCAO for the three experimental groups (Fig. 3C). Two-way ANOVA shows a statistical significant difference between groups ((F(1.854,59.32) = 3.780 and p = 0.0315)) and post-MCAO days ((F(3,32) = 23.47 and p = 0.0001)), but there was no significant interaction between these factors ((F(6,64) = 0.9123 and p = 0.4920)). Although young adult male group have a higher mean body weight change on post-MCAO day 7, Tukey’s multiple comparisons test did not show a statistically significant difference between groups on any post-MCAO days (p > 0.05). Likewise, mean grip strength decreased following MCAO for all three groups on post-stroke days 1 and 3 (Fig. 3D). The middle-aged male group showed persistent decrease in grip strength measurement on all days following MCAO. Two-way ANOVA shows a statistically significant difference between measured timepoints ((F(1.848,14.78) = 5.615 and p = 0.0169)) with lack of significant difference between groups ((F(1.427,11.42) = 3.954 and p = 0.0605)) as well as no significant interaction between these factors ((F(2.409,19.27) = 2.282 and p = 0.1213)).

Adhesive label removal time increased above baseline values for all groups across all measured timepoints (Fig. 3E), although with no statistical significance (p > 0.05). Time of adhesive label removal retuned to near baseline level for middle aged females on day 7, when compared to the male groups. Consequently, two-way ANOVA reveals a statistically significant difference between measured timepoints ((F(2.506, 60.15) = 16.86 and p = 0.0001)) with lack of significant difference between groups ((F(2, 24) = 0.9753 and p = 0.3915)) as well as no interaction between these factors ((F(6, 72) = 0.9905 and p = 0.4383)).

Similarly, vibrissae score on the impaired side was persistently decreased from baseline for the three groups, on all measured time points (Fig. 3F). Two-way ANOVA demonstrates a statistically significant difference between measured timepoints ((F(2.278, 54.68) = 57.61 and p = 0.0001)) with lack of significant difference between groups ((F(2, 24) = 0.4668 and p = 0.6326) as well as no interaction between these factors ((F(6, 72) = 1.098 and p = 0.3722)).

## Discussion

The present study provides evidence that the 0.43 mm monofilament size is more appropriate for over-weight (> 500 g) middle-aged male SD rats when compared with 0.45 mm and 0.47 mm Doccol^®^ monofilaments, and on the basis of CBF measures, infarct volume, body weight changes and functional outcomes. Additionally, the present study also analyzed the appropriate monofilament sizes for commonly observed weight ranges of middle-aged male (< 500 g) and female (> 350 g) SD rats.

Infarct volume is one of the predictors of stroke severity and outcome in patients [22–24]. To develop potential therapies for ischemic stroke, monofilament MCAO is an optimal preclinical model owing to the induction of large hemispheric infarct volume which is observed in humans [25, 26]. In study 1, while only 0.43 mm monofilament shows a statistically significant increase in infarct volume when compared to 0.47 mm (Fig. 1A), 0.43 mm and 0.45 mm monofilaments can induce large cerebral infarct volume following 90 min of MCAO in SD rats weighing > 500 g (Fig. 1B). However, the 0.43 mm group have a higher mean infarct volume when compared to the other two monofilament groups. Doccol^®^ suggested the use of 0.47 mm monofilament for animals weighing 501-600 g, but only one animal had observable infarct lesion of all the eleven (> 500 g) middle-aged male SD rats we tested using the 0.47 mm Doccol^®^ monofilament, in study 1. However, 0.43 mm monofilaments indicated for lighter animals weighing 401-500 g resulted in significantly larger infarct volume in these over-weight SD rats.

We ascertain that the difference in infarct volume between the three groups is not due to MCAO induction technique, as we observed a comparable percentage CBF reduction across all the groups. The present study reports pre-reperfusion CBF expressed as a percentage change from pre-MCAO (baseline) as all rats were allowed to recover from isoflurane anesthesia and move freely in a clean cage during MCAO and prior to reperfusion. Several reasons informed this choice. Firstly, we sought to prevent the unwanted effects of excess exposure to anesthesia. Isoflurane is a potent vasodilator and may increase CBF to the occluded MCA territory, hence confounding outcomes due to its neuroprotective effects [27–30]. Also, the monofilament may be dislodged from the origin of the MCA, potentially allowing blood to perfuse the territory in awake rodents during MCAO [31] and requiring a confirmation of CBF reduction prior to reperfusion. Additionally, this strategy affords the opportunity to observe rodent behavior and welfare during MCAO. Although pre-reperfusion CBF did not statistically differ between all three monofilament groups in study 1 (Fig. 2A), our results demonstrate a mean of ≥ 70% CBF reduction prior to reperfusion in all three groups when compared to baseline CBF. Several other studies reported setting the minimum CBF reduction immediately after MCAO necessary to induce infarct to be 60% [32–35], 85% [36] and 70% being the most frequently used cut off [37–39]. While the findings of the present study, even at pre-reperfusion CBF values, is in consonance with these studies, a recent study did not find a statistical correlation between 70% CBF reduction and infarct volume after MCAO in rodents [31].

In Study 2, we analyzed the infarct volume following 90 min of transient MCAO and 7-days post-surgery survival in young adult male, middle aged male and female SD rats which fall within the common weight ranges per age and sex group (Fig. 3). We did not observe statistically significant differences in infarct volumes between groups on post-stroke day 7 (Fig. 3A). However, the middle-aged female group had a relatively smaller mean infarct volume when compared with the two male SD groups. This result is similar to the infarct volume differences observed in other experimental studies [40–42]. In young female rats, the levels of endogenous estrogen influences infarct size [43]. As such, younger animals with regular estrous cycles have smaller infarct sizes while aged female animals with infrequent estrous cycles have larger infarct volumes [44]. Indeed, regular cyclicity is indicative of robust estrogen levels whereas acyclicity from middle age is characterized by persistent estrous or diestrus as well as very low or undetectable blood estrogen concentrations [45–47]. Although we did not measure blood estrogen concentration in the present study, we confirmed that all the middle-aged female SD rats used for this study were at diestrus phase on the day of MCAO induction and brain harvest. Despite this indication of irregular cyclicity and reduced estrogen protection, we observed the unexplainable smaller infarct volume in these middle-aged SD rats. Further studies need to explore the mechanistic factors responsible for the observed smaller infarct volume reduction at midlife in female SD rats.

Due to the established knowledge of variability in infarct volume with the monofilament model [16] as well as observations that infarct sizes increase in SD rats with increasing duration of MCAO from 60 to 75 min, [48] in the present study we increased our occlusion time to 90 min in order to ascertain the maximum brain infarct possible without impacting the post-MCAO welfare of the SD rats. Yet, our results show variability in infarct volume in both study 1 (Fig. 1A) and study 2 (Fig. 3A). Using the MCAO model, the observed variability in infarct volume may partly arise from the reported atypical branching of the MCA specific to SD rats. One study found that 20% of SD rats have atypical branching of the MCA [49] which may allow blood flow to some areas of the MCA territory with proximal intraluminal filament MCAO model as used in the present study. The chances of atypical MCA branching may increase with inbreeding, as the SD rats used for this study are from an inbred colony. Interestingly, there are also rat strain differences on infarct formation in experimental stroke. In one study [50], Wistar rats had larger infarct lesion than SD rats following transient and permanent MCAO. This indicates that duration of MCAO in SD rats is an important consideration. Here, we show that 90 min transient MCAO is adequate to demonstrate malignant ischemic stroke in both 24 h and 7 days survival cohorts of SD rats.

Ischemic stroke causes extensive brain damage which results in sensory and motor deficits [51]. Therefore, the determination of functional outcome is of great translational value in any preclinical ischemic stroke report. Study 1 of the present report reveals that 0.43 mm monofilaments significantly reduce neurological function, indicated by the 10-point neuroscore test, when compared with the 0.47 mm size. However, we observed a comparable mean functional deficit between 0.43 mm and 0.45 mm groups, but 0.43 mm had a lower mean neuroscore indicating worse outcome. Decreases in body weight generally correlate with a change in physiology and welfare which is directly associated with functional outcome after stroke. We show that the 0.43 mm group had substantial mean body weight loss when compared to other groups and that the difference between 0.43 mm and 0.47 mm groups is statistically significant. Overall, the neurological and body weight observations show that 0.43 mm monofilament is a more appropriate diameter for intraluminal monofilament model of MCAO in overweight middle aged SD rats.

Additionally, in study 2 the statistical differences in grip-strength and vibrissae-evoked forelimb placement tests between measured time points (prior to and following MCAO) indicates that the filament sizes are appropriate per age and sex groups. However, we did not observe any statistically significant difference in adhesive label removal time for all measured timepoints when compared with baseline. One possible explanation for this could be insufficient baseline training sessions to allow the rats learn how to quickly remove the adhesive label. Even so, the indicated filament sizes per group induced somatosensory deficits in these rats as evident by the trend of increased latency to remove on post-surgery days 1 and 3 as well as the near return to baseline on post-surgery day 7.

We did not observe statistical differences in weight changes, grip-strength test, vibrissae-evoked forelimb placement test, and sticky dot test across all three animal groups, which further shows that the selected monofilament sizes are appropriate for the induction of MCAO.

Intraluminal monofilament MCAO model of ischemic stroke is both complex and challenging to induce; as it requires enormous human and capital resources. Also, the resulting infarct volume and functional deficits depend on heterogeneous factors, including but not limited to body weight, age, sex and co-morbidities. Selecting appropriate filament sizes is the critical first step to ensure successful induction of MCAO and subsequent improvement of translational therapeutic development in order to aptly justify the time and efforts committed to this area of research. Taken together, the present study fills gaps in knowledge with regard to the appropriate Doccol^®^ monofilament size for over-weight (> 500 g) middle aged rats as well as middle aged male and female SD rats with normal weight (< 500 g). This study is also subject to some limitations. We did not test multiple filament sizes for each animal group in Study 2 to provide alternative options for optimization. Also, in study 2, we did not assess the daily estrus cycle in middle-aged female SD rats to determine whether other phases occurred between MCAO induction and brain harvest. Also, we did not perform any biochemical or histological assays to assess estrogen levels or neuronal degeneration across different study groups.

In conclusion, we demonstrate that 0.43 mm Doccol^®^ monofilament is appropriate in the induction of MCAO and functional deficits in over-weight middle-aged male SD rats. Also, we provide evidence that using 0.41 mm, 0.37 mm, and 0.39 mm for middle-aged male and female, and young-adult male SD rats, respectively, within common body weight ranges is appropriate. Our data has the potential to help improve the translatability as well as reproducibility of similar pre-clinical studies using the transient MCAO model, specifically in middle-aged SD rats.

## Data Availability

The corresponding author will make supporting data available upon request.

## References

[1] Benjamin EJ , Blaha MJ, Chiuve SE, Cushman M, Das SR, Deo R, de Ferranti SD, Floyd J, Fornage M, Gillespie C, Isasi CR, Jiménez MC, Jordan LC, Judd SE, Lackland D, Lichtman JH, Lisabeth L, Liu S, Longenecker CT, Mackey RH, Matsushita K, Mozaffarian D, Mussolino ME, Nasir K, Neumar RW, Palaniappan L, Pandey DK, Thiagarajan RR, Reeves MJ, Ritchey M, Rodriguez CJ, Roth GA, Rosamond WD, Sasson C, Towfighi A, Tsao CW, Turner MB, Virani SS, Voeks JH, Willey JZ, Wilkins JT, Wu JH, Alger HM, Wong SS, Muntner P, American Heart Association Statistics Committee and Stroke Statistics Subcommittee. Heart disease and stroke statistics-2017 update: a report from the American Heart Association [published correction appears in Circulation. 2017;135(10):e646 and 2017;136(10):e196]. Circulation. 2017;135(10):e146–e603. 10.1161/CIR.0000000000000485 PMC5408160 28122885

[2] Feigin VL, Lawes CM, Bennett DA, Anderson CS (2003). Stroke epidemiology: a review of population-based studies of incidence, prevalence, and case-fatality in the late 20th century. Lancet Neurol.

[3] Singhal AB, Biller J, Elkind MS, Fullerton HJ, Jauch EC, Kittner SJ (2013). Recognition and management of stroke in young adults and adolescents. Neurology.

[4] Roy-O'Reilly M, McCullough LD (2018). Age and sex are critical factors in ischemic stroke pathology. Endocrinology.

[5] Putaala J, Haapaniemi E, Kaste M, Tatlisumak T (2012). How does number of risk factors affect prognosis in young patients with ischemic stroke?. Stroke.

[6] Kissela BM, Khoury JC, Alwell K (2012). Age at stroke: temporal trends in stroke incidence in a large, biracial population. Neurology.

[7] Lisabeth LD, Baek J, Morgenstern LB, Zahuranec DB, Case E, Skolarus LE (2018). Prognosis of midlife stroke. J Stroke Cerebrovasc Dis.

[8] Mokin M, Ansari SA, McTaggart RA (2019). Indications for thrombectomy in acute ischemic stroke from emergent large vessel occlusion (ELVO): report of the SNIS Standards and Guidelines Committee. J Neurointerv Surg.

[9] Kleindorfer DO, Khoury J, Moomaw CJ (2010). Stroke incidence is decreasing in whites but not in blacks: a population-based estimate of temporal trends in stroke incidence from the Greater Cincinnati/Northern Kentucky Stroke Study. Stroke.

[10] Martinsen R, Kirkevold M, Sveen U (2015). Young and midlife stroke survivors' experiences with the health services and long-term follow-up needs. J Neurosci Nurs.

[11] Skolarus LE, Wing JJ, Morgenstern LB (2016). Mexican Americans are less likely to return to work following stroke: clinical and policy implications. J Stroke Cerebrovasc Dis.

[12] Vestling M, Tufvesson B, Iwarsson S (2003). Indicators for return to work after stroke and the importance of work for subjective well-being and life satisfaction. J Rehabil Med.

[13] Jorgensen HS, Nakayama H, Reith J (1997). Stroke recurrence: predictors, severity, and prognosis. The Copenhagen Stroke Study. Neurology.

[14] Pettersen R, Dahl T, Wyller TB (2002). Prediction of long-term functional outcome after stroke rehabilitation. Clin Rehabil.

[15] Macrae IM (2011). Preclinical stroke research–advantages and disadvantages of the most common rodent models of focal ischaemia. Br J Pharmacol.

[16] Percie du Sert N, Alfieri A, Allan SM, Carswell HV, Deuchar GA, Farr TD, Flecknell P, Gallagher L, Gibson CL, Haley MJ, Macleod MR, McColl BW, McCabe C, Morancho A, Moon LD, O’Neill MJ, Pérez de Puig I, Planas A, Ragan CI, Rosell A, Roy LA, Ryder KO, Simats A, Sena ES, Sutherland BA, Tricklebank MD, Trueman RC, Whitfield L, Wong R, Macrae IM (2017). The IMPROVE guidelines ischaemia models: procedural refinements of in vivo experiments. J Cereb Blood Flow Metab.

[17] Bouley J, Fisher M, Henninger N (2007). Comparison between coated vs uncoated suture middle cerebral artery occlusion in the rat as assessed by perfusion/diffusion weighted imaging. Neurosci Lett.

[18] Biose IJ, Dewar D, Macrae IM, McCabe C (2020). Impact of stroke co-morbidities on cortical collateral flow following ischaemic stroke. J Cereb Blood Flow Metab.

[19] Gileta AF, Fitzpatrick CJ, Chitre AS, St Pierre CL, Joyce EV, Maguire RJ, McLeod AM, Gonzales NM, Williams AE, Morrow JD, Robinson TE, Flagel SB, Palmer AA (2022). Genetic characterization of outbred Sprague Dawley rats and utility for genome-wide association studies. PLoS Genet.

[20] Koizumi JYY, Nakazawa T, Ooneda G (1986). Experimental studies of ischemic brain edema. A new experimental model of cerebral embolism in rats in which recirculation can be introduced in the ischemic area. Jpn J Stroke.

[21] Swanson RA, Morton MT, Tsao-Wu G, Savalos RA, Davidson C, Sharp FR (1990). A semiautomated method for measuring brain infarct volume. J Cereb Blood Flow Metab.

[22] Saver JL, Johnston KC, Homer D (1999). Infarct volume as a surrogate or auxiliary outcome measure in ischemic stroke clinical trials. The RANTTAS Investigators. Stroke.

[23] Helenius J, Henninger N (2015). Leukoaraiosis burden significantly modulates the association between infarct volume and National Institutes of Health Stroke Scale in ischemic stroke. Stroke.

[24] Ospel JM, Hill MD, Menon BK (2021). Strength of association between infarct volume and clinical outcome depends on the magnitude of infarct size: results from the ESCAPE-NA1 trial. AJNR Am J Neuroradiol.

[25] Carmichael ST (2005). Rodent models of focal stroke: size, mechanism, and purpose. NeuroRx.

[26] Mrosk F, Hecht N, Vajkoczy P (2021). Decompressive hemicraniectomy in ischemic stroke. J Neurosurg Sci.

[27] Bleilevens C, Roehl AB, Goetzenich A, Zoremba N, Kipp M, Dang J, Tolba R, Rossaint R, Hein M (2013). Effect of anesthesia and cerebral blood flow on neuronal injury in a rat middle cerebral artery occlusion (MCAO) model. Exp Brain Res.

[28] Zhou Y, Lekic T, Fathali N, Ostrowski RP, Martin RD, Tang J, Zhang JH (2010). Isoflurane posttreatment reduces neonatal hypoxic-ischemic brain injury in rats by the sphingosine-1-phosphate/phosphatidylinositol-3-kinase/Akt pathway. Stroke.

[29] Sullender CT, Richards LM, He F, Luan L, Dunn AK (2022). Dynamics of isoflurane-induced vasodilation and blood flow of cerebral vasculature revealed by multi-exposure speckle imaging. J Neurosci Methods.

[30] Hoffmann U, Sheng H, Ayata C, Warner DS (2016). Anesthesia in experimental stroke research. Transl Stroke Res.

[31] Morris GP, Wright AL, Tan RP, Gladbach A, Ittner LM, Vissel B (2016). A comparative study of variables influencing ischemic injury in the Longa and Koizumi methods of intraluminal filament middle cerebral artery occlusion in mice. PLoS ONE.

[32] Taninishi H, Jung JY, Izutsu M, Wang Z, Sheng H (2015). A blinded randomized assessment of laser Doppler flowmetry efficacy in standardizing outcome from intraluminal filament MCAO in the rat. J Neurosci Methods.

[33] Zhang J, Cao S, Kwansa H, Crafa D, Kibler KK (2012). Transfusion of hemoglobin-based oxygen carriers in the carboxy state is beneficial during transient focal cerebral ischemia. J Appl Physiol.

[34] Hall AA, Guyer AG, Leonardo CC, Ajmo CT, Collier LA (2009). Human umbilical cord blood cells directly suppress ischemic oligodendrocyte cell death. J Neurosci Res.

[35] Rowe DD, Leonardo CC, Recio JA, Collier LA, Willing AE (2012). Human umbilical cord blood cells protect oligodendrocytes from brain ischemia through Akt signal transduction. J Biol Chem.

[36] Liu F, Schafer DP, McCullough LD (2009). TTC, Fluoro-Jade B and NeuN staining confirm evolving phases of infarction induced by middle cerebral artery occlusion. J Neurosci Methods.

[37] Barber PA, Rushforth D, Agrawal S, Tuor UI (2012). Infrared optical imaging of matrix metalloproteinases (MMPs) up regulation following ischemia reperfusion is ameliorated by hypothermia. BMC Neurosci.

[38] Pignataro G, Simon RP, Xiong ZG (2007). Prolonged activation of ASIC1a and the time window for neuroprotection in cerebral ischaemia. Brain.

[39] Molinaro P, Cuomo O, Pignataro G, Boscia F, Sirabella R (2008). Targeted disruption of Na+/Ca2 + exchanger 3 (NCX3) gene leads to a worsening of ischemic brain damage. J Neurosci.

[40] Murphy SJ, McCullough LD, Smith JM (2004). Stroke in the female: role of biological sex and estrogen. ILAR J.

[41] Li K, Futrell N, Tovar S, Wang LC, Wang DZ, Schultz LR (1996). Gender influences the magnitude of the inflammatory response within embolic cerebral infarcts in young rats. Stroke.

[42] Sohrabji F, Okoreeh A, Panta A (2019). Sex hormones and stroke: beyond estrogens. Horm Behav.

[43] Carswell HV, Dominiczak AF, Macrae IM (2000). Estrogen status affects sensitivity to focal cerebral ischemia in stroke-prone spontaneously hypertensive rats. Am J Physiol Heart Circ Physiol.

[44] Selvamani A, Sohrabji F (2010). Reproductive age modulates the impact of focal ischemia on the forebrain as well as the effects of estrogen treatment in female rats. Neurobiol Aging.

[45] Huang HH, Steger RW, Bruni JF, Meites J (1978). Patterns of sex steroid and gonadotropin secretion in aging female rats. Endocrinology.

[46] LeFevre J, McClintock MK (1988). Reproductive senescence in female rats: a longitudinal study of individual differences in estrous cycles and behavior. Biol Reprod.

[47] Wise PM, Ratner A (1980). LHRH-induced LH and FSH responses in the aged female rat. J Gerontol.

[48] Encarnacion A, Horie N, Keren-Gill H, Bliss TM, Steinberg GK, Shamloo M (2011). Long-term behavioral assessment of function in an experimental model for ischemic stroke. J Neurosci Methods.

[49] Fox G, Gallacher D, Shevde S, Loftus J, Swayne G (1993). Anatomic variation of the middle cerebral artery in the Sprague-Dawley rat. Stroke.

[50] Walberer M, Stolz E, Müller C, Friedrich C, Rottger C, Blaes F, Kaps M, Fisher M, Bachmann G, Gerriets T (2006). Experimental stroke: ischaemic lesion volume and oedema formation differ among rat strains (a comparison between Wistar and Sprague-Dawley rats using MRI). Lab Anim.

[51] Tsao CW, Aday AW, Almarzooq ZI (2022). Heart disease and stroke statistics-2022 update: a report from the American Heart Association. Circulation.

